# Is radiotherapy necessary for intermediate risk ductal carcinoma in situ after breast conserving surgery?

**DOI:** 10.1186/2193-1801-3-405

**Published:** 2014-08-05

**Authors:** Taeryung Kim, Heung Kyu Park, Kyung Hee Lee, Kwan Il Kim, Kyu Chan Lee, Jeong Suk Ahn, Kwang-Pil Ko

**Affiliations:** Breast Cancer Center, Department of Surgery, Gachon University Gill Hospital, 1198, Guwol-dong, Incheon, 405-760 Korea; Breast Cancer Center, Department of Radiation Oncology, Gachon University Gill Hospital, Incheon, Korea; Breast Cancer Center, Department of Pathology, Gachon University Gill Hospital, Incheon, Korea; Department of Preventive Medicine, Gachon University Graduate School of Medicine, Incheon, Korea

**Keywords:** Breast cancer, Ductal carcinoma in situ, Radiotherapy, Van Nuys Prognostic index, Recurrence, ER

## Abstract

Identifying ductal carcinoma in situ (DCIS) patients at highest risk for recurrence after breast conserving surgery (BCS) remains a clinical concern. Subjecting all such patients to radiotherapy may be unnecessary. The Van Nuys Prognostic Index (VNPI) is a simple scoring system for predicting the risk of local recurrence in patients with DCIS. We reviewed patients with DCIS applying the VNPI score system. A total of 184 DCIS patients who underwent surgery at our institution between January 2003 and December 2011 were identified. Patients were not treated according to VNPI guidelines; rather, radiation therapy was applied at each surgeon’s discretion. All patients with hormonal receptor positive tumors were treated with hormonal therapy. Pathology reports were reviewed and VNPI scores of each DCIS calculated. Of the 184 patients, 52 (28.3%), 115 (62.5%) and 17 (9.2%) had low, intermediate and high VNPI scores, respectively. Six of the 184 patients (3.3%) developed ipsilateral local recurrence, five in the intermediate and one in the high VNPI score group. Of the five in the intermediate group, three (60%) were in patients with ER-negative tumors. VNPI score itself was not associated with recurrence (P = 0.145). Factors associated with recurrence included tumor size (hazard ratio [HR] 6.88), grade (HR 9.07) and hormone receptor status (HR 11.75). Radiotherapy did not significantly improve recurrence rates in patients with low and intermediate risk DCIS, especially in those with ER-positive tumors. Radiotherapy can be omitted in patients with ER-positive intermediate score DCIS and in patients with low score DCIS.

## Introduction

DCIS of the breast encompasses a heterogeneous spectrum of diseases, characterized by the proliferation of cancer cells within the ducts without invasion of the surrounding stromal tissue (Harris et al. 1992). Although little is known regarding the natural history of DCIS, patients with DCIS have a potential risk of invasive cancer (Robinson et al. 2008); therefore its management is similar to that for early invasive breast cancer (Masson and Bahl 2013). Studies of DCIS treated with excision reported 5-year local recurrence (LR) rates of 6–15% (Ottesen et al. 2000; Schwartz et al. 1992; Baird et al. 1990). Due to the risk of recurrence following breast conserving surgery (BCS), patients with DCIS frequently receive adjuvant radiotherapy (RT). RT following surgery may reduce the LR rate by about 50%, and a meta-analysis of four randomized studies comparing wide excision with and without RT showed that RT significantly reduced recurrence (odds ratio [OR] 0.40; 95% confidence interval [CI] 0.33-0.60, p < 0.00001) (Viani et al. 2007).

At the time of those studies, however, pathologic factors related to local control were largely unknown, resulting in underestimation of invasiveness and margin status. Although BCS followed by RT has become the standard of management in DCIS, several retrospective series have suggested that adjuvant RT may be omitted for patients at lower risk for recurrence. Identifying patient subgroups likely to show local control without additional RT is therefore important, since RT has various side effects, ranging from skin reactions to radiation pneumonitis. Pulmonary toxicities include lung cancer and pulmonary fibrosis (persistent cough or breathlessness), which negatively affect patient quality of life. Thus, the benefits of RT with respect to local control must be compared with its risks of side effects.

Historical data on tumor recurrence have led to the development of tools predicting the need for adjuvant treatment, with the Van Nuys Prognostic Index (VNPI) being the most widely used. The VNPI was developed from a retrospective analysis of a large database of patients with DCIS (Silverstein et al. 1996). Factors included in the VNPI are tumor size, grade, surgical margin and patient age, resulting in scores ranging from 4 to 12 (Table 1). Patients at low risk can undergo excision only, those at intermediate risk require adjuvant RT following BCS and those at high risk require mastectomy. The present study investigated outcomes in patients with DCIS who underwent BCS with or without RT at our institution. We also analyzed the results using the VNPI scoring system.

**Table 1:** **Van Nuys Prognostic index, modified from Silverstein MJ; DCIS of the breast 2nd ed. 2002**

Score	1	2	3
**Size (mm)**	≤15	16-40	≥41
**Margin width (mm)**	≥10	1-9	<1
**Pathology**	Grade 1/2 without necrosis	Grade 1/2 with necrosis	Grade 3 with or without necrosis
**Age**	>60	40-60	<40

## Patients and methods

A review of our institution’s medical records identified 184 patients with DCIS who underwent surgery between January 2003 and December 2011. Patients with a prior history of cancer and those with DCIS with microinvasion were excluded. Clinicopathologic characteristics evaluated included patient age, tumor size, grade, margin status, and tumor biology, and adjuvant therapies were also noted.

Surgical treatment options were BCS or mastectomy, depending on various clinical factors. Although all pathology reports included VNPI scores, these scores were not regarded as absolute in selecting treatment. If surgical margin status was close or positive, re-excision or total mastectomy was performed. No patient underwent RT after mastectomy. The RT dose to the wholebreast was 50.4 Gy/28 fx, with tumor bed boost given to all RT patients with 10 Gy/5 fx. Patients with hormone receptor positive tumors were treated with adjuvant hormonal therapy.

### Statistical analysis

Data were statistically analyzed using SPSS version 18 (SPSS Inc, Chicago, IL). Continuous variables were compared using variance analysis and categorical variables were compared using the chi-square test. Disease free survival (DFS) was defined as the interval from the date of surgery to the date of recurrence. DFS was estimated by the Kaplan-Meier method and compared using the log-rank test. Cox proportional hazards regression models were used to assess unadjusted hazard ratios (HRs), with the latter calculations performed using SAS (SAS Institute, Cary, NC) software. A p value < 0.05 was defined as statistically significant.

## Results

Patient characteristics are shown in Table 2. The mean age was 49 years, with 12% aged <40 years and 75% aged 41–60 years. Mean tumor size was 24.8 mm, with 76% of the tumors being of low to intermediate grade and 72.8% being ER-positive. Of the 184 patients, 127 (69.0%) underwent BCS and 57 (31.0%) underwent total mastectomy (TM). In addition, 31 (16.8%) patients underwent no axillary procedure, 109 (59.2%) underwent sentinel lymph node biopsy only, and 44 (23.9%) underwent axillary lymph node dissection. Margin widths were determined after the final excision in the patients who underwent BCS, with 21 (11.4%) undergoing re-excision for safety margins. Margins >9 mm were attained in 50 patients (39.4%), final margins 1–9 mm in 59 patients (46.5%), and final margins <1 mm in 18 patients (14.2%). Most patients (90.8%) had low to intermediate VNPI scores. Of the 127 patients who underwent BCS, 38 (29.9%) had low risk, 75 (59.1%) had intermediate risk, and 14 (11%) had high risk VNPI scores (Table 3). Of the 57 patients who underwent TM, 14 (24.6%) had low risk and 43 (75.4%) had intermediate risk VNPI scores. None of the patients in the TM group had a high VNPI score, since the margins after TM were estimated to be ≥10 mm. All patients with high risk VNPI scores underwent postoperative RT, whereas those with low and intermediate VNPI scores underwent postoperative RT at the discretion of the surgeon. All hormone receptor positive patients were treated with tamoxifen.

**Table 2:** **Characteristics of patient with DCIS (n=184)**

Variable	Value
**Age (yr)**	48.9±9.5
**Operation**	
**BCS**	127(69%)
**Total mastectomy**	57(31%)
**Tumor size (mm)**	
**≤15**	65(35.3%)
**16-40**	66(35.9%)
**≥41**	53(28.8%)
**Tumor grade**	
**1**	32(17.4%)
**2**	108(58.7%)
**3**	44(23.9%)
**Margin width**	
**<1**	18(9.8%)
**1-9**	68(36.9%)
**≥10**	98(53.3%)
**Receptor status**	
**ER positive**	134(72.8%)
**ER negative**	50(27.2%)
**PR positive**	116(63.0%)
**PR negative**	68(37.0%)
**VNPI score**	
**Low (4-6)**	52(28.3%)
**Intermediate (7-9)**	115(62.5%)
**High (10-12)**	17(9.2%)

**Table 3:** **Characteristics of patients with and without recurrence in breast conserving group**

Variable	Recurrence (n=6)	No recurrence (n=121)	*P*
**Age (yr)**	46.2±6.9	48.5±8.9	.697
**≤40**	1(16.7%)	17(14.0%)	
**41-60**	5(83.3%)	91(75.2%)	
**≥61**	0	13(10.7%)	
**Tumor size (mm)**	51.4±28.0	23.5±19.1	.000
**≤15**	0	58(47.9%)	
**16-40**	1(16.7%)	42(34.7%)	
**≥41**	5(83.3%)	21(17.4%)	
**Tumor grade**			.033
**1**	0	28(23.1%)	
**2**	2(33.3%)	67(55.4%)	
**3**	4(66.7%)	26(21.5%)	
**Margin (mm)**	10.6±8.6	9.4±7.6	.314
**>9**	4(66.7%)	46(38.0%)	
**1-9**	2(33.3%)	57(47.1%)	
**<1**	0	18(14.9%)	
**Multifocality**			.201
** Yes**	2(33.3%)	16(13.2%)	
** No**	4(66.7%)	105(86.8%)	
**Receptor status**			.031
**ER positive**	2(33.3%)	94(77.7%)	
**ER negative**	4(66.7%)	27(22.3%)	
**PR positive**	2(33.3%)	83(68.6%)	.092
**PR negative**	4(66.7%)	38(31.4%)	
**C-erbB2**			.354
**0**	0	16(13.2%)	
**1+**	0	31(25.6%)	
**2+**	3(50%)	28(23.1%)	
**3+**	3(50%)	44(36.4%)	
**Unknown**	0	2(1.4%)	
**Ki67 (%)**			.700
**<10**	2(33.3%)	65(53.7%)	
**10-19**	2(33.3%)	22(18.2%)	
**≥20**	2(33.3%)	31(25.6%)	
**VNPI score**			.260
**4-6**	0	38(31.4%)	
**7-9**	5(83.3%)	70(57.9%)	
**10-12**	1(16.7%)	13(10.7%)	
**Tamoxifen**	2(33.3%)	93(76.9%)	.035
**Radiation**	6(100.0%)	89(73.6%)	.336

**Figure 1:**
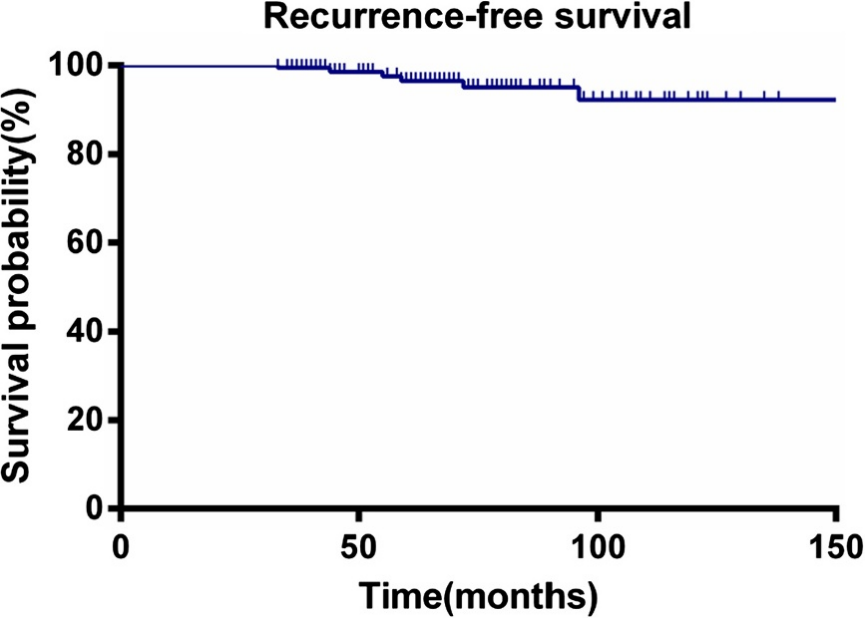
**Ipsilateral breast tumor recurrence (IBTR)-free survival.**

**Figure 2:**
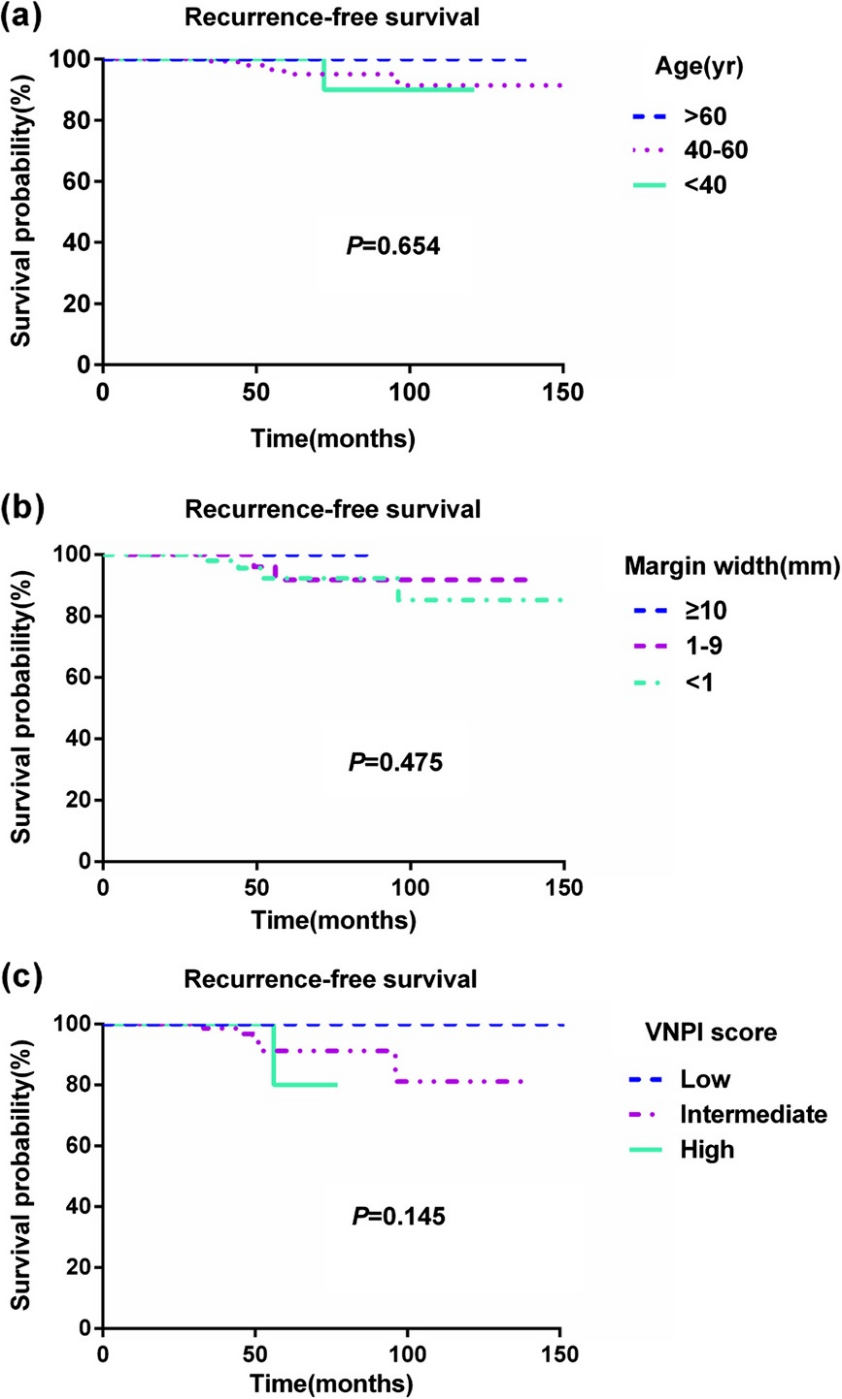
**Ipsilateral breast tumor recurrence(IBTR)-free survival. (a)** IBTR-free survival according to age **(b)** IBTR-free survival according to margin width **(c)** IBTR-free survival according to VNPI score.

Median follow-up period was 66 months. Six of the 184 patients (3.3%) developed ipsilateral local recurrence 33 to 96 months postoperatively (median, 50.5 months). Recurrence profiles are shown in Table 4. The 6-year ipsilateral breast tumor recurrence (IBTR)-free survival rate was 96.7% (Figure 1). Three of the recurrences were invasive (50%). No patient died during the follow-up period. None of the patients in the TM group experienced a tumor recurrence, compared with 6 of 127 in the BCS group. Five of six recurrences occurred in patients with intermediate VNPI scores, with the sixth in a patient with a high VNPI score, the latter 47 months postoperatively. This patient was a 42 year old woman with an ER negative tumor 47 mm in size of grade 3; the patient had a VNPI score of 10 and received postoperative RT. Statistically significant differences in tumor size, grade, and ER status were observed between patients with and without recurrence (Table 3). Univariate analysis using the Kaplan-Meier log rank test showed that tumor size (HR 6.88), grade (HR 9.07) and hormone receptor status (HR 11.75) were associated with recurrence (Tables 5 and 6). ER status was a greater predictor of recurrence than tumor size or grade. Univariate analysis using a Cox regression model showed that RT was not associated with recurrence (HR = 11.41; 95% CI 0.475-273.931, P =0.1334). Age, margin width, Her-2 status and Ki-67 were not significantly associated with DFS. Similarly, when VNPI scores were used to predict recurrence, there were no statistically significant differences in recurrence rates among patients with low, intermediate and high scores (Figure 2). In addition, there was no relationship between VNPI and prognosis, regardless of age (data not shown).

**Table 4:** **Profile of patients with ipsilateral breast tumor recurrence**

No.	Age (yr)	Surgery	Size (mm)	Margin width (mm)	Pathology	VNPI score	ER/PR/Her2	RT	IBTR type	Time to recurrence (month)
1	51	BCS	42	25	G3 with necrosis	9	+/+/-	yes	Invasive	96
2	36	BCS	55	3	G2 without necrosis	9	-/-/+	yes	Invasive	49
3	45	BCS	50	10	G2 without necrosis	7	+/+/-	yes	Invasive	52
4	50	BCS	95	10	G3 with necrosis	9	-/-/-	yes	DCIS	44
5	42	BCS	47	5	G3 with necrosis	10	-/-/+	yes	DCIS	56
6	52	BCS	18	10	G3 with necrosis	8	-/-/+	yes	DCIS	33

**Table 5:** **Clinical prognostic factors in BCS group(n=127), Kaplan Meier log-rank test**

Variable	Recurrence	No recurrence	Percentage	*P*value (log rank)
**Age (yr)**				.789
**≤40**	1	17	1/18(5.6%)	
**41-60**	5	91	4/96(4.2%)	
**≥61**	0	13	0	
**Tumor size (mm)**				<.001
**≤15**	0	58	0	
**16-40**	1	42	1/43(2.3%)	
**≥41**	5	21	5/26(19.2%)	
**Tumor grade**				.002
**1**	0	28	0	
**2**	2	67	1/69(1.4%)	
**3**	4	26	4/30(13.3%)	
**VNPI score**				.173
**4-6**	0	38	0	
**7-9**	5	70	5/75(6.7%)	
**10-12**	1	13	1/14(7.1%)	
**ER status**				.016
**(+)**	2	94	2/96(2.1%)	
**(-)**	4	27	4/31(12.9%)	
**RT**				.184
**Yes**	6	89	6/95(6.3%)	
**No**	0	32	0	

**Table 6:** **Factors associated with recurrence,univariate analysis using Cox regression model**

Variable	Univariate HR	*P*
**Age (yr)**		
**≤40**	1.32	.334
**41-60**	Ref	-
**≥61**	-	-
**Tumor size (mm)**		
**≤15**	-	**-**
**16-40**	Ref	**-**
** ≥41**	6.88	.001
**Tumor grade**		
** 1**	-	-
** 2**	Ref	-
** 3**	9.07	.003
**Margin (mm)**		
**>9**	1.39	.499
** 1-9**	Ref	-
**<1**	-	-
**Receptor status**		
** ER positive**	Ref	-
** ER negative**	11.75	.003
** C-erbB2**		
**≤2+**	Ref	-
**3+**	1.15	.284
**VNPI score**		
** 4-6**	-	-
** 7-9**	.417	.519
** 10-12**	Ref	-
**Radiation**		
** Yes**	Ref	-
** No**	1.77	.183

Most recurrences occurred in the intermediate VNPI score group. Of the 14 patients in this group (18.7%) who did not undergo RT, none showed evidence of tumor recurrence. In contrast, recurrences occurred in patients who received RT, with 3 of 5 (60%) occurring in patients with ER-negative tumors. A retrospective data review showed that tumor size was significant in determining treatment options among patients with intermediate VNPI scores. Surgeons have a tendency to add RT or to perform a mastectomy based on tumor size rather than margin width (Table 7). However, adding RT did not affect local control of disease.

**Table 7:** **Clinicopathologic characteristics in VNPI intermediate scores according to treatment (TM; total mastectomy in intermediate VNPI score)**

Variable	No RTx (n=14)	RTx (n=61)	TM (n=43)	*P*
**Age (yr)**	49.1±9.5	48.1±7.9	48.7±9.5	.979
**≤40**	2(14.3%)	7(11.7%)	6(14.0%)	
**41-60**	11(78.6%)	50(82.0%)	33(76.7%)	
**≥61**	1(7.1%)	4(6.6%)	4(9.3%)	
**Microcalcification**				.983
**Yes**	9(64.3%)	38(62.3%)	28(65.1%)	
**No**	5(35.7%)	23(37.7%)	15(34.9%)	
**Multifocality**				.314
**Yes**	1(7.1%)	9(14.8%)	10(23.3%)	
**No**	13(92.9%)	52(85.2%)	33(76.7%)	
**Tumor size (mm)**	13.93±12.07	29.90±18.11	50.74±23.49	.001
**≤15**	11(78.6%)	14(23.0%)	1(2.3%)	
**16-40**	2(14.3%)	30(49.2%)	15(34.9%)	
**≥41**	1(7.1%)	17(27.9%)	27(62.8%)	
**Tumor grade**				.360
**1**	2(14.3%)	8(13.1%)	1(2.3%)	
**2**	7(50.0%)	36(59.0%)	28(65.1%)	
**3**	5(35.7%)	17(27.9%)	14(32.6%)	
**Margin (mm)**	8.93±7.97	7.80±7.58		.221
**>9**	5(35.7%)	18(29.5%)	-	
**1-9**	9(64.3%)	32(52.5%)	-	
**<1**	0	11(18.0%)	-	
**VNPI score**	7.5±0.7	8.2±0.8	8.0±0.9	.021
**Receptor status**				
**ER positive**	13(92.9%)	40(65.6%)	28(65.1%)	.110
**PR positive**	10(71.4%)	38(62.3%)	21(48.8%)	.240
**C-erbB2**				.636
**0**	3(21.4%)	6(9.8%)	6(14.0%)	
**1+**	2(14.3%)	10(16.4%)	11(25.6%)	
**2+**	3(21.4%)	18(29.5%)	6(14.0%)	
**3+**	6(42.9%)	26(42.6%)	18(41.9%)	
**Unknown**	0	1(1.7%)	2(4.7%)	
**Ki67 status (%)**	12.8±12.39	13.65±15.54	12.91±15.94	.847
**Tamoxifen**	12(85.7%)	43(68.9%)	30(69.8%)	.467
**Recurrence**	0	5(8.3%)	0	.418

## Discussion

Attempts to validate the VNPI scoring system have yielded mixed results (MacAusland et al. 2007; Di Saverio et al. 2008; Gilleard et al. 2008; Altintas et al. 2011; Lee et al. 2013; Whitfield et al. 2012; Asjoe et al. 2007). Such studies have had uneven population distributions, with most patients having low to intermediate scores. Although patient age, tumor size, and tumor grade were set at patient diagnosis, margin width may be downgraded by re-excision or TM. Such efforts to salvage treatment have resulted in a disproportionately low rate of patients with high VNPI scores (MacAusland et al. 2007; Di Saverio et al. 2008; Whitfield et al. 2012). A prospective trial with a short follow-up time found that, in the absence of RT or tamoxifen, downscoring the risk of recurrence made it impossible to analyze factors normally because of the small number of recurrences (Wong et al. 2006). In our study, however, the VNPI scoring system was not a treatment guideline, with patients receiving RT at the discretion of individual surgeons or patients. The proportion of patients who underwent TM in the intermediate risk group was relatively high (36.4%), and was only somewhat lower in the low risk group (26.9%), which may reflect patient preference, such as for a definitive cure.

About half of the IBTRs were invasive, similar to findings of approximately 50% invasive relapses after BCS in other studies (Schwartz et al. 1992; Lagios 1990; Nakamura et al. 2002; de Mascarel et al. 2000). The proportion of patients deemed at high risk was 11%, higher than in other studies. Most of the recurrences occurred in patients with intermediate scores, with only one occurring in the high risk group.

Of the 17 patients in the high risk group, 14 (82.4%) underwent BCS followed by RT, whereas only three patients underwent TM, with recurrences observed in one (7.1%) and zero patients, respectively. The benefits of RT could not be determined, however, due to a lack of comparison, since all RTs were performed in this group. A previous study, which recommended mastectomy in patients with high risk scores, evaluated VNPI retrospectively in only 333 patients, with their findings not considered evidence-based. This prognostic index can promote discussions between physicians and patients, based on tumor parameters obtained after local excision (Asjoe et al. 2007). Although the benefits of RT are unknown, the addition of RT to BCS should be compared clinically with mastectomy plus reconstruction.

We also found that none of the women with BCS who did not receive RT developed tumor recurrence. In contrast, most recurrences were in the intermediate risk group who received postoperative RT. As in other studies, we observed no “RT-effect” in our low risk group (Lee et al. 2013; Whitfield et al. 2012; Asjoe et al. 2007). Contrary to previous findings, no “RT-effect” was observed in our intermediate risk group. RT has been recommended in all DCIS patients at intermediate risk of recurrence. However, we found that RT did not decrease recurrence rate in some intermediate risk patients. That is, following excision, patients with intermediate risk DCIS could be safely ‘undertreated’ under some circumstances.

Interestingly, 60% of intermediate risk patients with recurrence had ER-negative DCIS. Of the 127 women in our study who underwent BCS, with or without RT, 31 (24.4%) were ER-negative and 96 (75.6%) were ER-positive, with the former more likely to experience local recurrence than the latter (9.7% vs 3.7%), or a 7.6% absolute difference. An evaluation of 132 patients with DCIS treated with BCS without (n = 33) or with (n = 99) whole-breast RT found an absolute difference of about 8.5% in rates of local recurrence in patients with ER-negative and ER-positive DCIS (Roka et al. 2004; Provenzano et al. 2003). Indeed, several studies have revealed an association between ER-negative DCIS and risk of recurrence (Ringberg et al. 2001; Provenzano et al. 2003; Kerlikowske et al. 2010).

Several randomized clinical trials may determine the ability of ER to predict response to endocrine therapy. For example, the NSABP B-24 trial, which included 1804 women and had a median follow-up time of 12 years, found very small but statistically significant reductions in rates of breast cancer events among patients randomized to tamoxifen versus placebo, including a 3.2% reduction in ipsilateral invasive recurrence, but no change for ipsilateral DCIS. However, in that study, patients were not selected to receive tamoxifen on the basis of ER positivity. The degree of benefit may have been higher if only patients with ER-positive DCIS had been enrolled. Taken together, these results suggest that ER is weakly predictive of local recurrence following treatment for DCIS (Lari and Kuerer 2011). In the present study, all women with ER-positive DCIS received tamoxifen, although their adherence was not evaluated. Of patients who underwent BCS, with or without RT, 75.6% are ER-positive, suggesting that patients with DCIS may benefit from endocrine therapy since none experienced recurrence regardless of RT. The finding that all recurrences were in patients with ER-negative DCIS indicates that recurrences are due to biologic characteristics of the tumor associated with ER negativity, not with RT. ER negativity was significantly also associated with a higher risk of recurrence than ER positivity (HR = 11.75; 95% CI 0.93-183.74, *P* value = 0.04) Since only 6 patients experienced recurrences, our analysis loses considerable discriminating power, and we were unable to study the independent contributions of each prognostic factor in multivariate analysis. Instead, we calculated HR and 95% CI using a univariate Cox regression model. Our results indicate the relatively greater importance of ER negativity compared to VNPI score. The VNPI score itself did not provide sufficient evidence to predict prognosis in our cohort.

The question remains whether TM is more beneficial in high risk patients with ER-negative DCIS. We found that the ER-negative DCIS rate was 47.1% in the high risk group, with 37.5% undergoing TM and 62.5% undergoing BCS with RT. None of the former, and one of the latter, experienced tumor recurrence, suggesting that mastectomy has a 1.25-fold higher benefit than BCS with RT. Patients with large, high grade, ER-negative DCIS may require TM, whereas those with ER-positive DCIS may be treated with BCS plus RT.

In the intermediate risk group, the ER-negative DCIS rate was 30.5%. Of patients in the intermediate risk group, 18.7% did not undergo RT, with almost all of these patients having ER-positive DCIS. All recurrences were observed in the RT group, including two (40%) with ER-positive and three (60%) with ER-negative DCIS. Adherence to tamoxifen treatment was not evaluated, since we assumed good compliance with hormonal therapy. The benefits of RT seemed to be greater in patients with ER negative than ER positive-DCIS, suggesting the importance of a local control strategy for ER-negative DCIS. In contrast, RT may be unnecessary in patients with ER-positive intermediate risk DCIS. Hormonal therapy alone may be sufficient, allowing patients to avoid the side effects of RT.

We observed no association between margin width and recurrence. In our study population, only 9.8% had margin widths <1 mm. The margin widths of most tumors are large enough after excision; therefore margin status was not correlated with any other outcomes (Di Saverio et al. 2008; Ringberg et al. 2001; Lim et al. 2014; Yi et al. 2012).

Studies have reported that Ki-67 expression rates and HER2 and other biological markers are prognostically significant markers in DCIS patients (Roka et al. 2004; Ringberg et al. 2001; Provenzano et al. 2003; de Roos et al. 2007; Menter et al. 2001; Barnes et al. 2005). The US National Cancer Institute has recently identified a critical need for investigation and validation of molecular factors to improve risk stratification of patients with DCIS, thus facilitating a determination of the optimal therapy for each individual patient (Allegra et al. 2010). Although we observed no association between HER2 expression and recurrence, HER2 expression and status were not evaluated by FISH. Similarly, data on Ki67 expression was not reliably evaluated.

This single institution retrospective analysis had a median follow-up of 61 months. Longer follow-up is needed to estimate long-term outcomes. Additionally, selection bias should be expected due to the heterogeneities of the study population.

In conclusion, our findings suggest that adjuvant RT can be omitted in some patients with intermediate risk DCIS. ER status is important for stratifying risk of recurrence. RT may be unnecessary in patients with ER-positive intermediate score DCIS. Endocrine treatment should be provided to all patients with ER-positive DCIS.

### Ethical standards

The study protocol was reviewed and approved by the Institutional Review Board of the Gachon University Gil Hospital. Because this study was performed using a total of 184 consecutive patients in our database, and involved no more than minimal risk for the subjects, the Institutional Review Board approved our request for the waiver of informed consent. Recommendations of the Declaration of Helsinki for biomedical research involving human subjects were also followed.
